# Comparative evaluation of apical micro leakage in root canals obturated with different bio ceramic sealers

**DOI:** 10.6026/973206300220980

**Published:** 2026-02-28

**Authors:** Niral Kotecha, Sadaf Fatma, Sandip D Patil, Priyanka Chandra, Prabhat Saxena, Vikram Karande, Ritik Kashwani

**Affiliations:** 1 Department of Conservative Dentistry and Endodontics, Government Dental College and Hospital, Jamnagar, Gujarat, India; 2 Department of Dentistry, Buddha Institute of Dental Sciences, Kanti Factory Road, Kankarbagh, Patna, Bihar, India; 3 Department of Conservative Dentistry and Endodontics, Government Dental College and Hospital, Jalgaon, Maharashtra, India; 4 Department of Conservative Dentistry and Endodontics, Kothiwal Dental College and Research Centre, Kanth, Moradabad, Uttar Pradesh, India; 5 Department of Endodontics, Dentarch Solutions, Greater Noida, Uttar Pradesh, India; 6 Department of Oral & Maxillofacial Surgery, D.Y. Patil Dental School, Lohegaon, Pune, Maharashtra, India; 7 Department of Oral Medicine and Radiology, School of Dental Sciences, Sharda University, Greater Noida, Uttar Pradesh, India

**Keywords:** Apical micro leakage, bioceramic sealers, BioRoot RCS, Meta CeraSeal, root canal obturation

## Abstract

Apical microleakage remains a major cause of endodontic failure, emphasizing the need for root canal sealers with superior sealing ability. This *in vitro* study comparatively evaluated apical microleakage in 200 single-rooted teeth obturated using BioRoot RCS and Meta CeraSeal bioceramic sealers. Standardized canal preparation and obturation were performed and apical microleakage was assessed using a dye penetration method under stereomicroscopic evaluation. Statistical analysis revealed no significant difference in mean apical microleakage between the two groups (p > 0.05), indicating comparable sealing performance. This study advances current knowledge by demonstrating that both BioRoot RCS and Meta CeraSeal provide equally effective apical sealing, supporting evidence-based selection of bioceramic sealers in clinical endodontics.

## Background:

Apical microleakage remains one of the most critical factors influencing the long-term success of root canal treatment. Despite advances in endodontic instrumentation and irrigation protocols, failure of endodontic therapy is frequently attributed to inadequate sealing of the root canal system, allowing the ingress of microorganisms, fluids and their by-products into the periapical tissues [1]. Achieving a three-dimensional hermetic seal is therefore considered a fundamental objective of root canal obturation, as it directly impacts periapical healing and prevention of reinfection [2]. Root canal sealers play a pivotal role in obturation by filling the interface between the core obturating material and the dentinal walls, as well as penetrating into accessory canals, lateral canals and dentinal tubules [3]. Conventional sealers, including zinc oxide eugenol-based, resin-based and calcium hydroxide-based sealers, have been widely used; however, each category exhibits inherent limitations such as shrinkage, solubility, cytotoxicity, or lack of bioactivity. These short comings have driven the development of newer materials with improved physicochemical and biological properties [4]. Bioceramic sealers represent a significant advancement in endodontic materials science. These sealers are primarily composed of calcium silicate-based compounds and are characterized by their excellent biocompatibility, hydrophilicity, dimensional stability and bioactivity [5]. Their ability to set in the presence of moisture, release calcium ions and form hydroxyapatite at the sealer-dentin interface enhances chemical bonding with dentinal walls and promotes biological sealing. Additionally, the alkaline pH of bioceramic sealers contributes to their antibacterial properties, further supporting periapical healing [6]. Among the bioceramic sealers available in the Indian clinical scenario, BioRoot RCS and Meta CeraSeal have gained considerable popularity due to their availability, ease of handling and favorable clinical performance.

BioRoot RCS is a tricalcium silicate-based sealer known for its bioactive potential, strong adhesion to dentin and ability to stimulate mineralization [7]. Meta CeraSeal, a premixed calcium silicate-based sealer, offers the advantage of convenient delivery, consistent composition and adequate flow characteristics, which may influence its penetration into dentinal tubules and sealing efficiency. Despite their increasing use, variations in formulation, setting reaction and interaction with dentin may result in differences in apical sealing ability [8]. Apical microleakage evaluation serves as an essential parameter for assessing the sealing efficacy of root canal sealers. *In vitro* studies employing dye penetration, fluid filtration, bacterial leakage, or microscopic analysis provide valuable insight into the sealing behavior of obturation materials under standardized conditions [9]. Comparative assessment of newer bioceramic sealers is particularly important, as clinical evidence is still evolving and material selection often relies on laboratory-based outcomes [10]. A direct comparison between BioRoot RCS and Meta CeraSeal with respect to apical microleakage can help clinicians make evidence-based decisions when selecting sealers for routine endodontic practice, especially in regions where these materials are commonly used. Understanding their sealing performance also contributes to improving obturation quality and long-term treatment success [11]. Therefore, it is of interest to evaluate the apical microleakage associated with root canals obturated using BioRoot RCS and Meta CeraSeal bioceramic sealers.

## Methodology:

This *in vitro* comparative study was conducted to evaluate apical microleakage in root canals obturated using two different bioceramic sealers-BioRoot RCS and Meta CeraSeal. A total of 200 freshly extracted human single-rooted permanent teeth were collected following ethical clearance and informed consent for use in research. Teeth extracted for periodontal or orthodontic reasons were included, while those with cracks, fractures, resorption, calcifications, open apices, previous endodontic treatment, or anatomical variations were excluded. All specimens were cleaned of soft tissue remnants and calculus and stored in 0.1% thymol solution until use. Standardization of specimens was achieved by decoronating all teeth at the cementoenamel junction using a diamond disc under water cooling to obtain a uniform root length of approximately 15 mm. Working length was determined by inserting a size #10 K-file until it was visible at the apical foramen and subtracting 1 mm from this length. Root canal preparation was performed using rotary nickel- titanium instruments up to size F3, following the manufacturer's instructions. Irrigation was carried out using 3% sodium hypochlorite during instrumentation, followed by a final rinse with 17% EDTA to remove the smear layer and a final flush with distilled water. Canals were dried using sterile paper points. The prepared samples were randomly divided into two experimental groups of 100 teeth each. In Group I, root canals were obturated using gutta-percha in combination with BioRoot RCS sealer. In Group II, root canals were obturated using gutta-percha with Meta CeraSeal bioceramic sealer. In both groups, the sealers were applied according to the manufacturers' instructions and obturation was performed using the single-cone technique. Excess gutta-percha was removed and the coronal access was sealed with temporary restorative material. All specimens were stored at 37°C with 100% humidity for 7 days to allow complete setting of the sealers. Following setting, the external root surfaces were coated with two layers of nail varnish, except for the apical 2 mm, to restrict dye penetration to the apical region. The samples were then immersed in 2% methylene blue dye solution for 24 hours. After dye exposure, teeth were rinsed under running water, dried and longitudinally sectioned in a buccolingual direction using a diamond disc. Apical microleakage was evaluated under a stereomicroscope at 20x magnification by measuring the linear extent of dye penetration from the apex toward the coronal direction. Measurements were recorded in millimeters using calibrated imaging software. The collected data were subjected to statistical analysis using appropriate tests to compare apical microleakage between the two groups, with the level of significance set at p < 0.05.

## Results:

The present *in vitro* study evaluated apical microleakage in 200 root canal-treated teeth obturated using two bioceramic sealers: BioRoot RCS (Group I) and Meta CeraSeal (Group II), with 100 samples in each group. All specimens were available for analysis and no sample loss was recorded during the experimental procedure. Descriptive statistical analysis revealed comparable apical microleakage values between the two groups. Group I (BioRoot RCS) demonstrated a mean dye penetration of 1.24 ± 0.31 mm, whereas Group II (Meta CeraSeal) showed a mean value of 1.27 ± 0.34 mm. The distribution of microleakage values in both groups was found to be approximately normal, permitting the use of parametric statistical tests. The overall comparison suggested minimal variation in sealing ability between the two bioceramic sealers (Table 1). An independent sample t-test was performed to compare the mean apical microleakage values between the two groups. The analysis revealed no statistically significant difference between BioRoot RCS and Meta CeraSeal (p = 0.48), indicating that both sealers exhibited comparable sealing efficacy at the apical level (Table 2). Further analysis of microleakage severity was carried out by categorizing dye penetration values into three ranges: ≤1.0 mm, 1.1-1.5 mm and >1.5 mm. Both groups demonstrated a similar distribution pattern across these categories, with the majority of samples falling within the 1.1-1.5 mm range. This distribution further supports the comparable performance of the two sealers in limiting apical dye penetration (Table 3). Intra- group variability was assessed using standard deviation and coefficient of variation. Both groups demonstrated low variability, suggesting consistent sealing performance within each material. BioRoot RCS showed a coefficient of variation of 25.0%, while Meta CeraSeal demonstrated a slightly higher but comparable value of 26.8% (Table 4). To evaluate the reliability of measurements, inter-observer agreement was assessed using intraclass correlation coefficient (ICC). The ICC value of 0.91 indicated excellent agreement between observers, confirming the reliability and reproducibility of the microleakage measurements across both experimental groups (Table 5). Overall, statistical analysis confirmed that both BioRoot RCS and Meta CeraSeal bioceramic sealers provided effective and comparable apical sealing, with no material demonstrating superiority over the other in terms of microleakage.

## Discussion:

The findings of the present study showing comparable apical microleakage between BioRoot RCS and Meta CeraSeal sealers align with emerging evidence that many bioceramic sealers provide effective sealing in root canal obturation. In our study, no statistically significant difference was observed in apical dye penetration between the two materials, suggesting that both sealers confer similar apical sealing efficacy. This is consistent with results reported by Singhal *et al.* (2025) [11], who compared the apical sealing ability of BioRoot RCS, CeraSeal and another bioceramic sealer using a dye penetration model after immersion in simulated body fluid. They found that although initial microleakage varied among groups, after 30 days of exposure to body fluid there was no significant difference in microleakage among the bioceramic sealers tested, indicating comparable sealing potential across these materials when used with a single-cone technique. In a similar in vitro assessment, Sachin *et al.* (2024) [12] compared CeraSeal and BioRoot RCS with an epoxy resin sealer using a dye-extraction leakage method. They reported no significant difference in microleakage between the two bioceramic sealers, although both showed slightly higher leakage compared with the resin-based control. This corroborates our finding that bioceramic sealers perform comparably to each other in terms of apical sealing. While the present study focused specifically on BioRoot RCS and Meta CeraSeal, Sah *et al.* (2024) [13] compared multiple endodontic sealers, including bioceramics and conventional materials and found that although sealers differed in their degree of microleakage, none demonstrated absolute impermeability. Their work reinforces the idea that improvements in material chemistry may reduce leakage, but complete elimination of microleakage remains elusive, even among bioceramic materials. Related aspect of sealing efficacy is penetration into dentinal tubules, which contributes to mechanical interlocking and sealing performance. Mann *et al.* (2025) [14] compared dentinal penetration of several calcium silicate-based sealers and reported that CeraSeal and BioRoot exhibited high penetration depth, suggesting enhanced sealing potential at the microscopic level. Although penetration depth does not directly equate to apical microleakage, it supports the premise that bioceramic sealers like those studied here can form intimate contact with dentin, which may underlie their similar leakage profiles. Taken together, these previous investigations support our findings that bioceramic sealers perform comparably with respect to apical microleakage when used in standardized obturation protocols. The cumulative evidence suggests that, within the limitations of *in vitro* models, neither BioRoot RCS nor Meta CeraSeal offers a distinct advantage over the other, aligning with broader observations in the endodontic literature. Further high-quality clinical studies are warranted to determine whether these *in vitro* outcomes translate into differences in long-term clinical success.

## Conclusion:

Within the limitations of this *in vitro* study, both BioRoot RCS and Meta CeraSeal bioceramic sealers demonstrated comparable apical sealing ability, with no statistically significant difference in microleakage. The results indicate that both materials provide effective apical seals when used with a standardized obturation technique. Therefore, either bioceramic sealer may be considered a reliable option for achieving optimal apical sealing in routine endodontic practice.

## Figures and Tables

**Table 1:** Descriptive statistics of apical microleakage (mm) in both groups

**Group**	**n**	**Mean (mm)**	**Standard Deviation**
BioRoot RCS	100	1.24	0.31
Meta CeraSeal	100	1.27	0.34

**Table 2:** Comparison of mean apical microleakage between groups (Independent t-test)

**Comparison**	**Mean Difference**	**t-value**	**p-value**
BioRoot RCS vs Meta CeraSeal	0.03	0.7	0.48

**Table 3:** Distribution of samples based on dye penetration range

**Dye Penetration Range (mm)**	**BioRoot RCS n (%)**	**Meta CeraSeal n (%)**
≤1.0	32 (32%)	30 (30%)
1.1-1.5	46 (46%)	48 (48%)
>1.5	22 (22%)	22 (22%)

**Table 4:** Intra-group variability of apical microleakage

**Group**	**Mean (mm)**	**SD**	**Coefficient of Variation (%)**
BioRoot RCS	1.24	0.31	25
Meta CeraSeal	1.27	0.34	26.8

**Table 5:** Inter-observer reliability for microleakage assessment

**Parameter**	**ICC Value**	**Interpretation**
Apical microleakage measurement	0.91	Excellent agreement
